# Kidney Is Essential for Blood Pressure Modulation by Dietary Potassium

**DOI:** 10.1007/s11886-020-01359-1

**Published:** 2020-08-13

**Authors:** Xiao-Tong Su, Chao-Ling Yang, David H. Ellison

**Affiliations:** 1 School of Medicine, Oregon Health and Science University, Portland, OR USA; 2 Oregon Clinical & Translational Research Institute, SN4N, Oregon Health and Science University, 3181 SW Sam Jackson Park Road, Portland, OR 97239 USA; 3 Veterans Administration Portland Health Care System, Portland, OR USA

**Keywords:** High-potassium diet, Blood pressure, Hypertension, Natriuresis, DCT, NCC

## Abstract

**Abstract:**

Eating more potassium may reduce blood pressure and the occurrence of other cardiovascular diseases by actions on various systems, including the vasculature, the sympathetic nervous system, systemic metabolism, and body fluid volume. Among these, the kidney plays a major role in the potassium-rich diet–mediated blood pressure reduction.

**Purpose of Review:**

To provide an overview of recent discoveries about the mechanisms by which a potassium-rich diet leads to natriuresis.

**Recent Findings:**

Although the distal convoluted tubule (DCT) is a short part of the nephron that reabsorbs salt, via the sodium-chloride cotransporter (NCC), it is highly sensitive to changes in plasma potassium concentration. Activation or inhibition of NCC raises or lowers blood pressure. Recent work suggests that extracellular potassium concentration is sensed by the DCT via intracellular chloride concentration which regulates WNK kinases in the DCT.

**Summary:**

High-potassium diet targets NCC in the DCT, resulting in natriuresis and fluid volume reduction, which are protective from hypertension and other cardiovascular problems.

## Introduction

Hypertension is a worldwide health problem affecting 40% of the population over the age of 25 [1]. A new assessment by the Global Burden of Disease consortium indicates that unless better approaches can be devised, hypertension will remain the predominant factor contributing to risk-attributable years of life lost in 2040 [2]. While multiple therapeutic strategies have been developed to treat hypertension, challenges still exist, with many patients remaining poorly controlled. High blood pressure usually does not cause symptoms, but it is one of the most common risk factors for non-communicable diseases and is a leading cause of healthy life loss, making it second to smoking as a preventable cause of mortality [3]. Hypertension is one of the strongest risk factors for cardiovascular diseases, including coronary disease, left ventricular hypertrophy, valvular heart disease, cardiac arrhythmias, cerebral stroke, and kidney failure. Lifestyle and nutrition are important factors that modulate blood pressure. Guideline-driven initial management of hypertension or pre-hypertension emphasizes non-pharmacological approaches, such as increasing physical activity, losing body weight, decreasing alcohol consumption, reducing sodium intake, and stopping tobacco smoking [4–6].

### Dietary Sodium and Hypertension

Among all nutritional factors, recommendations are mainly focused on the reduction of dietary sodium intake. Epidemiological and clinical studies have demonstrated associations between salt intake and blood pressure [7]. High-sodium diet is known to aggravate hypertension [8, 9] and increase cardiovascular diseases incidence [10–16]. Nevertheless, dietary sodium restriction activates the renin-angiotensin-aldosterone system (RAAS) and sympathetic nervous system (SNS) [17–19], which may counteract some purported benefits. Sympathetic nervous system hyperactivity has been recognized as a hallmark of progressive heart disease and congestive heart failure [20]. Although SNS activation is a compensatory protective mechanism in the short term, chronic activation has been shown to produce adverse effects on the cardiovascular system and may accelerate disease progression [20, 21]. For instance, peripheral stimulation of the sympathetic nerves of the failing heart may lead to ventricular arrhythmias [22]. Angiotensin II (AngII) affects both physiological processes and pathophysiological factors, many of which are critical in cardiovascular diseases, including vascular tone, cellular function, and cell growth [23–25]. AngII itself can contribute to fibrosis, endothelial cell dysfunction, thrombosis, and atherosclerosis [25–27]. These countervailing effects have led to continuous debates during the past 50 years about the “optimal” dietary salt intake, and debates that continue today [8, 28].

### Dietary Potassium and Blood Pressure

Other than sodium, many dietary constituents are included in the recommendations for healthy nutrition in patients at risk for hypertension, such as potassium, calcium, proteins, and magnesium. Both sodium and potassium are essential nutrients to help maintain fluid volume and cell structure. The typical diet consumed by many people in industrialized societies usually contains high amounts of sodium with less potassium, which differs from the Paleolithic diet, in which this ratio is reversed [29]. Most of potassium’s beneficial effects appear to be related to sodium, rather than an isolated response [30]. Most of the population consumes well above the recommended daily allowance of dietary sodium, and less than recommended potassium. Potassium is one of the four major shortfall nutrients (potassium, calcium, iron, and magnesium) in the western diet according to the 2015 Dietary Guidelines for American’s Advisory Committee [31]. Potassium intake has been related inversely to blood pressure and the occurrence of cardiovascular diseases [32]. This inverse relation has been further supported by large epidemiologic studies as well as smaller controlled trials [29, 30, 33–35]. Many suggest that higher potassium intake attenuates salt-sensitivity [36, 37], an effect corroborated in animal models [38]. The beneficial effects potassium may not, however, be uniform; recent animal studies and a meta-analysis of randomized-controlled trials suggest that excessive potassium intake may increase blood pressure [39, 40•]. The most impressive pressure-lowering effects of potassium are consistently observed when dietary salt consumption is also high.

### Potassium Effects on Systems Outside the Kidney

One mechanism by which high dietary potassium intake is reported to lower blood pressure is through vasodilatory effects. High dietary potassium stimulates the potassium channel, Kir2.1, and the Na/K pump in the vascular smooth muscle cell membrane, both of which tend to hyperpolarize the cell [41–43]. Stimulation of the Na/K pump decreases the intracellular sodium content, so that the sodium calcium exchanger type 1 (NCX1) favors calcium efflux, leading to vasodilation. Besides the direct vasodilation effects on vascular smooth muscle cells, high-potassium diet opens potassium channels and stimulates the Na/K pump in endothelial cells [42]. The endothelial hyperpolarization is transmitted to the vascular smooth muscle cell via myoendothelial gap junctions and by intracellular calcium sparks, which activates calcium-activated potassium channels [44]. This way, vascular smooth muscle cells are hyperpolarized indirectly, which leads to endothelium-dependent vasodilation. In addition to the vascular tone, high dietary potassium inhibits atherosclerosis and medial hypertrophy of the arterial wall [45, 46].

Brain and its interstitial fluid potassium content is tightly regulated and fluctuations in cerebrospinal fluid (CSF) potassium concentration ([K^+^]) may initiate responses to maintain CSF [K^+^] [47]. A sensing region of the brain near the ventricles can respond to changes in the sodium and potassium concentrations in the cerebrospinal fluid and regulates blood pressure [48, 49]. Increasing the potassium in the CSF by intraventricular potassium administration reduces blood pressure, whereas increasing sodium raises it [47, 50]. The central actions of potassium and sodium changes are mediated by altering the Na/K pump, and the effects are significantly attenuated by prior ouabain (Na/K pump inhibitor) central administration [50]. Antagonizing adrenergic and dopaminergic effects also blunted the potassium-induced blood pressure and heart rate reduction, indicating that central administration of potassium lowers the SNS outflow.

Hyperkalemia increases insulin secretion by depolarizing pancreatic beta cells, whereas hypokalemia inhibits insulin secretion and is associated with glucose intolerance [51, 52]. Insulin stimulates skeletal muscle blood flow by nitric oxide–mediated vasodilation and contributes to insulin sensitivity and responsiveness [53]. Compared with potassium-wasting diuretics, other classes of anti-hypertensive agents, including ACE inhibitors and calcium channel blockers, have a lower risk of insulin resistance, glucose intolerance, and onset of diabetes mellitus [54]. Potassium supplementation to treat diuretic-induced hypokalemia may reverse glucose intolerance and prevent the future development of diabetes [55].

### Potassium Effects on Blood Pressure Via the Kidney

Blood pressure is modulated by the nervous system, by vascular tone, through effects of baroreceptors and chemoreceptors and via cardiac output [56]. Over the long term, however, blood pressure regulation requires a balance between salt and water intake and output. The kidney is the major organ determining the salt and water output. This is exemplified by chronic kidney disease, in which small increases in extracellular fluid volume lead to blood pressure increases, which are often responsive to diuretics [57].

The kidney is the major organ responsible for electrolyte homeostasis. Unbound potassium and sodium are freely filtered across the glomerulus, and about 90% of the filtered load is reabsorbed along the proximal tubule and thick ascending limb [58]. This relation holds under most physiological conditions, so that final excretion is primarily determined in the distal nephron. Classical models focused on the role of the collecting duct in secreting potassium into the lumen [59]. Yet, it has more recently become clear that upstream segments, such as the distal convoluted tubule (DCT), the portion of the nephron immediately downstream of the macula densa, and the connecting tubule (CNT), play unique and important roles [60]. This is also the site along which fine control of sodium excretion is fulfilled via regulated sodium transport in kidney tubule segments beyond the macula densa, the first of which is the DCT [61]. Although the DCT is the shortest segment of the nephron, spanning only about 5 mm in length in humans [62], it is now recognized as a critical site in a variety of homeostatic processes, including sodium chloride reabsorption, potassium secretion, and calcium and magnesium handling. Potassium secretion begins in the late DCT and progressively increases along the distal nephron into the cortical collecting duct (CCD) via electrogenic potassium channels. Under normal conditions, the late DCT and CNT are the most pivotal in potassium secretion, whereas the CCD is critical primarily when animals are stressed or have high aldosterone levels [63].

The DCT reabsorbs roughly 5–7% of the filtered sodium load [64]. The electroneutral sodium-chloride cotransporter (NCC; *SLC12A3*) in the apical membrane is chiefly responsible for this process. Gitelman syndrome is the most common inherited tubular disease and results from mutations in the *SLC12A3* gene encoding NCC [65]. Patients with Gitelman syndrome exhibit potassium wasting, hypokalemia, hypomagnesemia, hypocalciuria, and hypovolemia-induced elevated angiotensin II and aldosterone levels, but they tend to have normal or even low blood pressure [66–68]. On the other hand, heterozygous mutations in NCC may prevent hypertension and cardiovascular diseases [69].

Recently, the DCT has been identified as a critical site for potassium homeostasis, although potassium secretion is not observed in the early DCT [70•, 71]. Regulation of NaCl reabsorption rates by NCC in the DCT is essential in adjusting the rate of potassium excretion. Patients with less NCC activity (Gitelman syndrome) exhibit kidney potassium wasting and hypokalemia, whereas patients with activated NCC, as occurs in the disease familial hyperkalemic hypertension (Gordon syndrome or pseudohypoaldosteronism type 2), exhibit decreased kidney potassium excretion and hyperkalemia. Clearly, mammals without normal regulation of NCC activity cannot maintain normal potassium balance, and a primary physiologic role of NCC is in potassium homeostasis [38]. Therefore, NCC is crucial in regulating electrolyte homeostasis, extracellular volume, and blood pressure.

### Potassium Switch [72]

The natriuretic effect of potassium in humans was reported more than 80 years ago [73]. Later, the blood pressure–lowering effects of potassium supplementation were also reported [14, 74, 75]. Although the beneficial effects of high potassium intake involve the vasculature, circulating factors, and the sympathetic nervous system, it has become clear recently that an essential mechanism is via natriuresis [76, 77]. Within the kidney, raising the paracellular potassium concentration has long been known to inhibit salt and fluid reabsorption along the proximal tubule [72] and the thick ascending limb [78]. It has been suggested recently that such proximal effects contribute substantially to the natriuretic effects of high potassium intake [79].

**Fig. 1:**
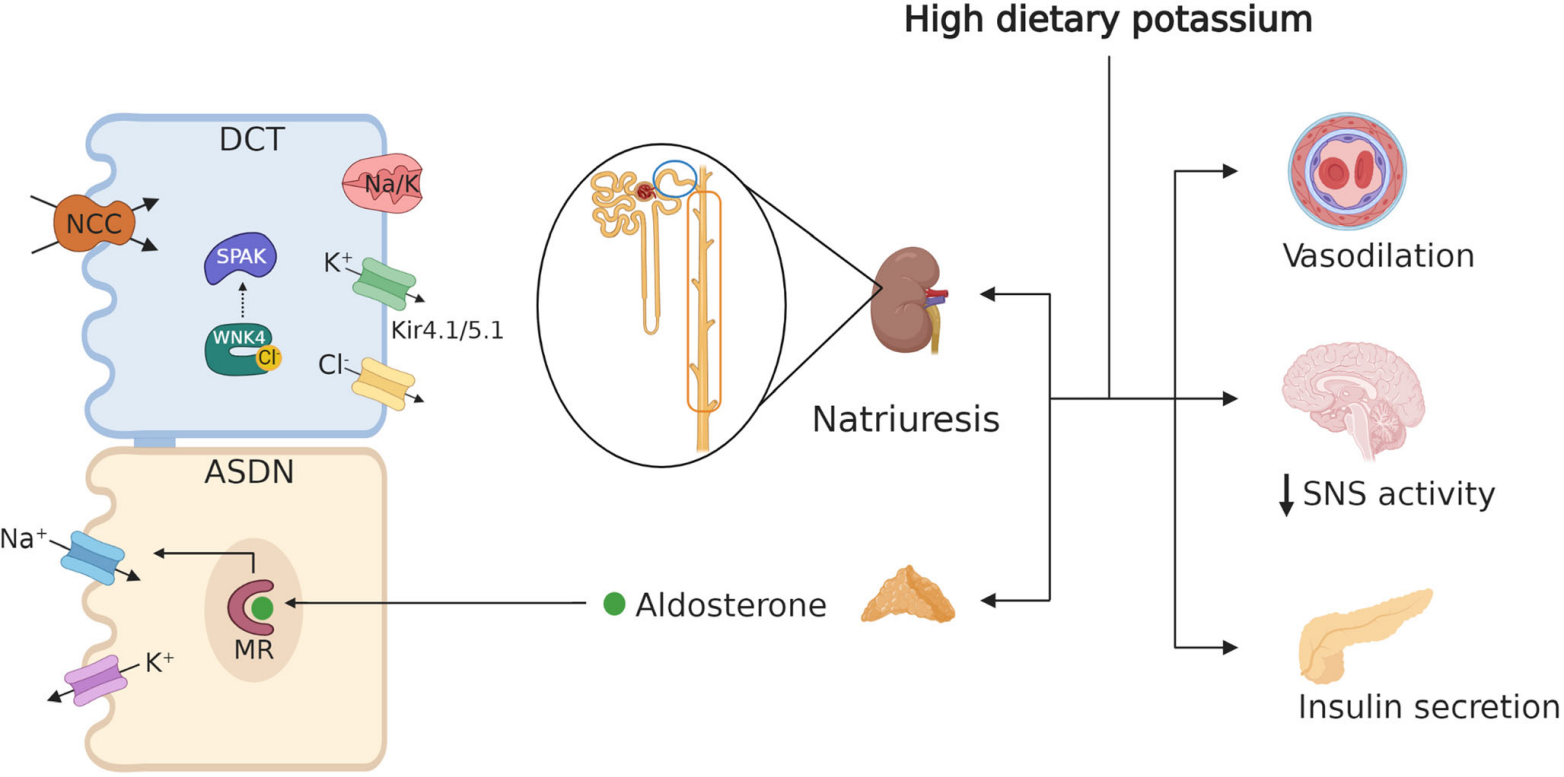
Pathways mediating beneficial effects of high potassium intake on various systems. High potassium intake increases insulin and aldosterone secretion and reduces vascular tone, sympathetic nervous system (SNS) outflow, and body fluid volume. In the kidney, high plasma potassium leads to inhibition of sodium-chloride cotransporter (NCC) in distal convoluted tubule (DCT) and increase of potassium secretion in aldosterone-sensitive distal nephron (ASDN), contributing to the natriuresis effects (see text for details)

Recently, however, a “kidney potassium switch” within the distal nephron has been identified as playing a dominant role in modulating sodium and potassium balance [38, 70•, 71, 80, 81]. This switch turns NCC off and apical epithelial sodium channel (ENaC) on in response to high potassium intake. An acute rise in plasma [K^+^] in the physiological range after a meal dephosphorylates NCC within minutes [82]. Terker et al. demonstrated an inverse linear relationship between phosphorylated NCC (pNCC, abundance of pNCC is a proxy for NCC activity) and plasma [K^+^] across a range of plasma [K^+^] manipulated by various factors [83]. During the past several years, it has become clear that the NCC in the DCT plays a unique role in determining kidney potassium excretion because of its specific site of expression and its unique mechanisms of regulation. Alterations in NaCl reabsorption in the DCT change the rate of Na^+^ delivery to the aldosterone-sensitive distal nephron (ASDN) which, when coupled with changes in aldosterone secretion, modulate electrogenic sodium reabsorption and potassium secretion. High potassium intake inhibits NCC and increases salt delivery to the ASDN. This may reduce water reabsorption via aquaporin-2 along the CNT. This leads to increased flow, which activates the apical epithelial sodium channel (ENaC) and increases the lumen negativity, facilitating potassium secretion. Additionally, high plasma [K^+^] stimulates aldosterone secretion, which increases ENaC activity in the ASDN [84]. The NCC inhibition–induced sodium wasting cannot be fully counteracted by the increase of downstream sodium reabsorption; thereby, high potassium intake reduces blood pressure by natriuresis-related fluid volume decrease (Fig. 1).

Given that NCC does not transport potassium and little potassium is secreted via early DCT, it seems unexpected that NCC activity is sensitive to plasma [K^+^]. In the past two decades, several molecular regulators have been uncovered with critical roles in the regulation of NCC, including WNK kinases, their downstream targets (SPAK, the STE20/SPS1-related, proline/alanine-rich kinase; and OSR1, the oxidative stress-responsive kinase 1), and cullin 3 and kelch-like 3 which participate in WNK degradation [85]. WNKs activate NCC by phosphorylating and activating SPAK/OSR1, which directly phosphorylates NCC along its amino terminal domain. Phosphorylation of NCC activates and stabilizes the transport protein, leading to increased solute transport [86, 87]. Yet, there is little evidence that WNKs or SPAK/OSR1 is sensitive to potassium.

Piala et al. reported that chloride binds and regulates WNK activity [88], supporting the hypothesis that chloride and WNKs serve as the secondary messengers to regulate apical membrane NaCl transport. At a higher intracellular concentration, chloride binds to the WNK catalytic domain and prevents kinase autophosphorylation and activation [88–92]. The intracellular chloride concentration ([Cl^−^]_i_) is dependent on the rate of chloride entry into and exit out of cells. In the DCT, chloride is transported transcellularly by the apical NCC and basolateral chloride channel (ClC-Kb) and potassium chloride cotransporter (KCC). The main driving force for chloride to exit the cell through the ClC-Kb is the negative membrane voltage. It has been shown previously that deletion of the basolateral potassium channel (Kir4.1/5.1) locks the DCT cells at a depolarized state and reduces the basolateral chloride channel conductance through an allosteric mechanism, which in turn eliminates the NCC response to plasma potassium alterations [70•, 71]. Modulating the basolateral potassium conductance affects chloride efflux, thereby should modulate [Cl^−^]_i_. Sun et al. showed that increases in extracellular [K^+^] raised [Cl^−^]_i_ and inhibited WNK activity in *Drosophila* Malpighian tubules [91•]. Terker et al. showed that low extracellular [K^+^] decreases [Cl^−^]_i_ and increases NCC phosphorylation and activation in transfected HEK cells, an effect that was blunted by mutating the WNK1 chloride binding site [38]. Chloride-insensitive WNK4 knock-in mice have increased NCC expression and activity, which is not responsive to low dietary potassium intake [93•]. These studies indicate that the chloride-dependent WNK activity is critical in the kidney potassium switch.

## Conclusions

Our ancestors in the Paleolithic era consumed a high-potassium and low-sodium diet (about 11,000 mg/day potassium and 700 mg/day sodium) with a ratio of 16:1 [94]. Consequently, the human body developed kidney mechanisms to excrete significant loads of potassium rapidly and to preserve sodium. The processed food we eat today contain more sodium and less potassium than natural food. In light of the recognition of its beneficial effects, dietary potassium recommendations were increased in 2004 when the recommended intake was established at 4700 mg/day [95]. The average potassium intake of Americans is just over half of this amount, 2591 mg/day. Regarding the potassium supplementation form, KHCO_3_ results in a greater cellular potassium uptake, a lower steady-state plasma potassium, and a lesser decrease in intracellular sodium concentration compared with KCl [96]. Although high potassium intake is protective from hypertension and a wide array of other cardiovascular problems, it is important to note that these beneficial effects have primarily been linked to eating high-potassium diets, rather than taking potassium supplements [97]. Fruits and vegetables are good sources of natural potassium and may have a greater blood pressure–lowering effect. We will benefit from switching away from processed food and embracing a Paleolithic diet rich in fruits and vegetables.
